# *Aggregatibacter actinomycetemcomitans* Omp29 Is Associated with Bacterial Entry to Gingival Epithelial Cells by F-Actin Rearrangement

**DOI:** 10.1371/journal.pone.0018287

**Published:** 2011-04-29

**Authors:** Mikihito Kajiya, Hitoshi Komatsuzawa, Annatoula Papantonakis, Makoto Seki, Seicho Makihira, Kazuhisa Ouhara, Yutaka Kusumoto, Shinya Murakami, Martin A. Taubman, Toshihisa Kawai

**Affiliations:** 1 Department of Immunology, Forsyth Institute, Boston, Massachusetts, United States of America; 2 Department of Oral Medicine, Infection and Immunity, Harvard School of Dental Medicine, Boston, Massachusetts, United States of America; 3 Department of Oral Microbiology, Kagoshima University Graduate School of Medical and Dental Sciences, Kagoshima, Japan; 4 Laboratory of Immunology, Faculty of Pharmacy, Osaka Ohtani University, Osaka, Japan; 5 Division of Oral Biology and Disease Control, Department of Periodontology, Osaka University Graduate School of Dentistry, Osaka, Japan; Tulane University, United States of America

## Abstract

The onset and progressive pathogenesis of periodontal disease is thought to be initiated by the entry of *Aggregatibacter actinomycetemcomitans* (*Aa*) into periodontal tissue, especially gingival epithelium. Nonetheless, the mechanism underlying such bacterial entry remains to be clarified. Therefore, this study aimed to investigate the possible role of *Aa* outer membrane protein 29 kD (Omp29), a homologue of *E. coli* OmpA, in promoting bacterial entry into gingival epithelial cells. To accomplish this, Omp29 expression vector was incorporated in an OmpA-deficient mutant of *E. coli*. Omp29^+^/OmpA^−^ *E. coli* demonstrated 22-fold higher entry into human gingival epithelial line cells (OBA9) than Omp29^−^/OmpA^−^ *E. coli*. While the entry of *Aa* and Omp29^+^/OmpA^−^ *E. coli* into OBA9 cells were inhibited by anti-Omp29 antibody, their adherence to OBA9 cells was not inhibited. Stimulation of OBA9 cells with purified Omp29 increased the phosphorylation of focal adhesion kinase (FAK), a pivotal cell-signaling molecule that can up-regulate actin rearrangement. Furthermore, Omp29 increased the formation of F-actin in OBA9 cells. The internalization of Omp29-coated beads and the entry of *Aa* into OBA9 were partially inhibited by treatment with PI3-kinase inhibitor (Wortmannin) and Rho GTPases inhibitor (EDIN), both known to convey FAK-signaling to actin-rearrangement. These results suggest that Omp29 is associated with the entry of *Aa* into gingival epithelial cells by up-regulating F-actin rearrangement via the FAK signaling pathway.

## Introduction

*Aggregatibacter actinomycetemcomitans* (*Aa*) is a putative pathogen of localized aggressive (juvenile) periodontitis [1], and the pathogenicity of this microorganism, especially its involvement in periodontal bone destruction, was demonstrated in a rat oral infection model [2]. Cytolethal distending toxin (CDT) and leukotoxin are postulated as virulent factors of *Aa* [3], [4]. Localization of *Aa* in the human gingival tissue was apparently demonstrated by immuno-fluorescent and immuno-histochemical analyses [5], [6]. It is plausible that the entry of *Aa* into gingival epithelium [7], [8] may facilitate the delivery of these toxins to host tissues. It was reported that *Aa* enters KB cells (human oral epidermal carcinoma) by promoting a rearrangement of host cytoskeletal components, such as actin microfilaments [9], indicating that entry of *Aa* is a consequence of bacterial uptake by epithelial cells and not bacterial intrusion.

The primary stimulus necessary for the cellular internalization of *Aa* appears to be elicited by initial bacterial binding to the cell surface molecules expressed on epithelial cells [7]. Previously, we have reported that outer membrane protein 100 (Omp100) of *Aa*, which possesses high homology to YadA, an outer-membrane protein of *Yersinia enterocolitica*, is associated with bacterial adhesion to KB cells or normal human gingival keratinocytes [10]. Although Omp100 can facilitate bacterial adhesion, an additional molecule of *Aa*, i.e., one that can act on the epithelial cells to elicit the uptake (entry) of the bacterium, is required. While it is plausible that one of the other major Omps of *Aa*, including Omp64, Omp39, Omp29, Omp18, and Omp16 [11], may be engaged in the induction of bacterial uptake by epithelial cells, the precise Omp and its mechanism associated with the induction of bacterial entry into gingival epithelial cells remain to be elucidated.

*E. coli* outer membrane protein A (OmpA) plays a key role in the bacterial entry into brain microvascular endothelial cells [12] and intestinal epithelial cells [13]. Anti-OmpA antibody inhibits the entry of *E. coli* into brain microvascular endothelial cells, and OmpA deletion mutant *E. coli* loses the capacity to enter into microvascular endothelial cells [12]. OmpA of *Klebsiella pneumoniae* is also capable of binding and activating human macrophages, consequently inducing the production of proinflammatory factors [14]. Importantly, our previous study revealed that Omp29 of *A. actinomycetemcomitans* belongs to the *E. coli* OmpA family [15]. Interestingly, contrary to Omp100, the gene encoding the OmpA/Omp29 family of molecules did not show homology to *Yersinia enterocolitica* YadA, suggesting a possibility that Omp29 has no role in bacterial adhesion to epithelial cells.

Based on these lines of evidence, we hypothesized that Omp29 is associated with the entry of *Aa* into gingival epithelial cells in a manner similar to *E. coli* OmpA. The results demonstrated that Omp29 does act on human gingival epithelial cells to induce their uptake of *Aa* by up-regulating the rearrangement of cytoskeleton fiber, F-actin.

## Materials and Methods

### Bacterial strains and culture

*Aa* strain Y4 (ATCC, Manassas, VA) was cultured in trypticase soy broth supplemented with 0.6% yeast extract (TSBY; Difco Laboratories, Detroit, MI) in humidified 5% CO_2_ atmosphere at 37°C. *Streptococcus sanguis* ATCC10556 (ATCC) was grown aerobically in TSBY. An OmpA-deficient mutant of *E. coli* (Bre51; *E. coli* K12 back ground) was a gift from Dr. Henning (Max-Planck-Institut für Biologie, Germany) [16]. *Aa* Omp16 and Omp29 were purified followingthe method previously published [11]. The Omp29 expression vector that contains the *Aa omp34* gene was incorporated in Bre51 by the method previously reported [15]. The *E. coli* Bre51 that expressed Omp29 was designated as *E. coli* 3826. *E. coli* 3826 and Bre51 were cultured at 37°C with Luria-Bertani (LB) broth containing ampicillin (100 µg/ml) when it was needed. Otherwise, both strains of *E. coli* were cultured with TSBY at 37°C. The bacterial number was measured by spectrophotometer at OD 580 nm.

### Primary culture and immortalized human gingival epithelial cell line and other epidermal carcinoma cell lines

The method of establishing an immortalized human gingival epithelial cell (HGEC) line (OBA9) from primary culture and its characteristics were previously published [17]. The primary cultures of HGEC and OBA9 cells were cultured with keratinocyte-serum free medium (K-SFM; Invitrogen, Buffalo, NY) in a plastic tissue flask pre-coated with type-I collagen (rat tail, BD Biosciences, Franklin Lakes, NJ). Epidermal carcinoma cell lines KB (ATCC) and Hep2 (ATCC) were maintained in DMEM (Invitrogen) supplemented with 10% FBS. The protocol to sample gingival tissue from periodontally healthy subject at the crown lengthening procedure was approved by the IRB of the Forsyth Institute. Informed consent was obtained before the collecting tissue sample.

### Polyclonal antibody specific to *Aa* Y4 Omp29

Polyclonal IgG antibodies to Omp29 or whole fixed *Aa* Y4 were developed in mice. BALB/c mice (8-week-old) were immunized (s.c.) with purified Omp29 (30 µg/animal) or formalin-fixed whole *Aa* Y4 (10^9^ cells/animal) in Freund's complete adjuvant, respectively. A second injection of each antigen in Freund's incomplete adjuvant and the booster injection of respective antigen in saline were carried out at two-week intervals from the initial immunization. Seven days after the booster injection, serum was collected from peripheral blood of euthanized mice. IgG in the serum was purified using a protein-G column purification kit (ImmunoPure IgG Purification kit; Pierce, Rockford, IL).

### Bacterial entry and adhesion to gingival epithelial cell lines

The confluent epithelial cells in a 96-well plate were cultured in antibiotic-free K-SFM overnight prior to the bacterial entry assay. Live bacteria harvested at mid-log growth curve were co-cultured with the epithelial cells in antibiotic-free K-SFM (5.5×10^5^ bacteria/100 µl/well) in the presence or absence of anti-Omp29 IgG or control non-immunized IgG. In some assays, phosphatidylinositol-3 (PI-3) kinase inhibitor Wortmannin (Biomol Research Laboratories, Plymouth Meeting, PA), a small GTP binding protein Rho inhibitor EDIN (epidermal cell differentiation inhibitor [18]), or actin rearrangement inhibitor cytochalasin D (Sigma, St. Louis, MO) was added to the medium. After appropriate time periods of co-culture, extracellular bacteria that did not enter the epithelial cells were killed by gentamicin (1 mg/ml in PBS; Sigma). The live bacteria remaining inside of the cells were released by osmotic disruption with distilled water (100 µl/well), followed by extensive pipetting (20 strokes/well). Bacterial suspension was spread onto TBSY agar plates (40 µl/plate), and the number of colonies was counted. The Colony Forming Unit (CFU) corresponds to the number of colonies observed in a TBSY agar plate. In order to count the adherent bacteria, the co-culture was disrupted and homogenized without gentamicin treatment containing both entered and adherent bacteria to epithelial cells. The number of adherent bacteria was calculated by subtracting the CFU obtained with gentamicin from the CFU obtained without gentamicin treatment.

### ELISA

The mouse IgG antibody response to Omp29 and fixed bacteria were evaluated by ELISA following the method previously described [19]. Purified Omp29 (1 µg/ml), or formalin-fixed whole *Aa* Y4, *E. coli* 3826 or *E. coli* Bre51 (10^6^/well, respectively) was coated onto a 96-well ELISA plate in sodium bicarbonate buffer (pH 9.4). Mouse anti-Omp29 IgG or control IgG was applied to each well, followed by biotin-labeled goat anti-mouse IgG (Boehringer Mannheim Biochemicals, Indianapolis, IN) and horseradish peroxidase (HRP)-conjugated streptavidin (Boehringer Mannheim). Colorimetric reaction was developed with o-Phenylenediamine (OPD; Sigma) in citrate buffer (pH 4.5) containing 0.02% H_2_O_2_. The developed color was measured by an ELISA reader at OD490 nm.

### SDS polyacrylamide gel electrophoresis (SDS-PAGE) and Western blot analysis

Purified Omp29 and bacterial samples were dissolved in SDS-PAGE loading buffer (Invitrogen) containing 2-mercaptoethanol and were boiled for 10 min. Proteins separated in 4–12% gradient SDS polyacrylamide gel (Invitrogen) were trans-blotted onto a nitrocellulose (NC) paper. The membrane was treated with blocking buffer (5% non-fat milk in phosphate buffered saline [PBS] containing 0.1% Tween 20, pH 7.4) for 1 hr at room temperature. The NC membrane was incubated with mouse anti-Omp29 IgG (1∶1000 dilution) overnight at 4°C. After reaction of membrane with HRP-conjugated donkey anti-mouse IgG antibody (Jackson Immunoresearch, West Grove, PA; 1∶5000), immunodetection was performed using Immobilon Western Chemiluminescent HRP substrate (Millipore, Billerica, MA).

Phosphorylation of focal adhesion kinase (FAK) in OBA9 cells was also determined by Western blot analysis. OBA9 cells were homogenized in a lysis buffer containing 25 mM Tris-HCl, 150 mM NaCl, 5 mM EDTA, 10% glycerol, 1% Triton X-100, 0.1% SDS, 1% NP-40, 1 mM PMSF, protease inhibitor cocktail (Sigma) and phosphatase inhibitor I and II (Sigma). The FAK protein in the cell lysate was immuno-precipitated with rabbit anti-total FAK monoclonal antibody (Cell Signaling, Beverly, MA; 1∶50) that was pre-bound to GammaBind Plus Sepharose™ beads (Pharmacia Biotech, Uppsala, Sweden). After extensive washing with the lysis buffer, proteins captured by the anti-FAK antibody coated beads were separated by SDS-PAGE and subjected to Western blot analyses with rabbit anti-phospho FAK (Tyr397) polyclonal antibody (Cell Signaling; 1∶500) or with rabbit anti-total FAK monoclonal antibody (1∶1000). After reaction of membrane with HRP-conjugated donkey anti-rabbit IgG (Jackson Immunoresearch, 1∶5,000), immunodetection was performed as described above.

### Immunofluorescence microscopy

Sub confluent OBA9 cells in 35 mm culture dish were pre-cultured in the presence or absence of anti-Omp29 IgG or control non-immunized IgG for 15 min. Then, cells were co-cultured with *Aa Y4* (1×10^7^ bacteria/2 ml/dish) in antibiotics free K-SFM for 1 hr and were fixed with 4% paraformaldehyde in PBS. Non-specific binding was blocked by incubation with PBS containing 1% BSA for 20 min. The cells were incubated with rabbit polyclonal anti-*Aa Y4* antibody (5 µg/ml) [11]at 4°C overnight. After three washes with PBS for 5 min, the cells were incubated with Aleaxa Fluor 594® anti-rabbit IgG (Invitrogen: 1∶200) and Alexa Fluor 488 phalloidin® (Invitrogen: 1∶250) for 1 hr at room temperature. Nuclei were stained with DAPI (Invitrogen: 5 µg/ml). After rinsing with PBS, fluorescence signals were detected with Olympus FSX100 fluorescence microscopy (Olympus, Tokyo, Japan).

### Detection of internalized fluorescent beads in OBA9 by flow cytometry

Omp29-coated fluorescent Latex beads (0.75 µm diameter; Polysciences Inc., Warrington, PA) were prepared following the method by Shimoji et al [20]. Briefly, fluorescent beads were coated with Omp29 in bicarbonate buffer (pH 9.6) for 2 hr at 27°C. Omp16- or BSA-coated beads were also prepared as control using the same method. The presence of Omp29 or Omp16 on each bead was confirmed by anti-Omp29 IgG or anti-whole *Aa* IgG. Confluent OBA9 in a 6-well plate was incubated with each bead suspension in the presence or absence of anti-Omp29 IgG (10 µg/ml), control IgG (10 µg/ml), Wortmannin (10 nM), or EDIN (100 ng/ml). After washing with PBS, OBA9 cells were removed from the culture plate by treatment with trypsin-EDTA. In order to remove beads that attached on the surface, but did not enter into OBA9 cells, the cell suspension was washed 5 times with cold PBS containing 0.02% EDTA. After the fourth wash, complete removal of beads from the cell surface was confirmed under fluorescent microscopy. The internalized beads in OBA9 were analyzed by a FACScan flow cytometer (Becton Dickinson, Mountain View, CA). Logarithmic amplification of fluorescent intensity on OBA9 (5,000 cells) was determined by forward light scatter intensity.

In order to analyze F-actin content of OBA9, confluent cells were incubated with or without purified Omp16 or Omp29 (10 µg/ml, respectively) in K-SFM. After the removal of OBA9 cells from the tissue culture plate by treatment with trypsin-EDTA, a single cell suspension of OBA9 was fixed with saline containing 5% formalin, and the cell membrane of OBA9 was permeabilized by 0.1% Tween 20 in PBS. F-actin in OBA9 was stained with FITC-conjugated phalloidin (×100 dilution, Sigma), and fluorescent intensity in each cell was collected by a flow cytometer.

## Results

### Entry and adhesion of *Aa* to OBA9

**Figure 1:**
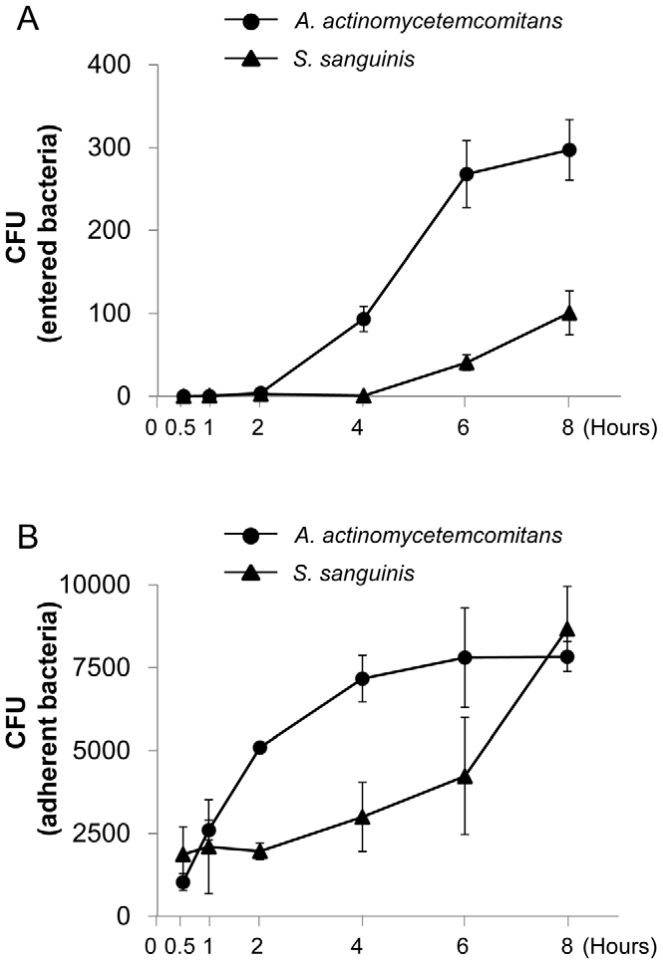
Kinetics of *Aa* or *S. sanguis* entry and adhesion to OBA9. (A and B) *Aa* or *S. sanguis* growing in middle of logarithmic phase in broth culture was applied to confluent OBA9 cells in 96-well plates (5.5×10^5^ bacteria/100 µl/well). The multiplicity of infection (MOI) value used in these entry and adhesion assays were about 55 (5.5×10^5^ bacteria/1.0×10^4^ epithelial cells/well). The co-culture of bacteria and OBA9 cells was incubated at various times, and bacterial entry and attachment to OBA9 cells were evaluated by the protocol described in Materials and Methods. The number of bacteria which entered (A) or adhered (B) to OBA9 was expressed as a colony forming unit (CFU). Each point represents mean ± SD of CFU from three wells.

In order to examine the properties of *Aa* Y4 to attach or enter to gingival epithelial cells, live *Aa* Y4 or control oral commensal bacterium, *S. sanguis*, was incubated with the confluent OBA9 cultured in 96-well plates for various periods (Fig. 1). The attachment of *Aa* to OBA9 occurred at an earlier incubation time (30 min) (Fig. 1B) than internalization (2 hours) (Fig. 1A). After 6–8 hours of incubation, both attachment and internalization of *Aa* Y4 reached saturation level. On the other hand, compared to *Aa* Y4, the initial entry event of *S. sanguis* was detected later incubation periods (6–8 hours) than *Aa* Y4, and the number of entered *S. sanguis* was remarkably lower than that of *Aa* Y4 during these late incubation periods (6–8 hours) (Fig. 1A). However, the adhesion of *S. sanugis* to OBA9 cells reached to the same level as *Aa* Y4 after 8 hr of incubation (Fig. 1B). The entry and attachment of *Aa* Y4 were also examined using different cell lines, including primary culture of human gingival epithelial cells (5^th^ passage) and epidermal carcinoma cell lines (KB and Hep2). *Aa* Y4 was capable of entering and adhering to all cell lines tested (data not shown). These findings suggested that a periodontal pathogen, *Aa* Y4, periodontal pathogenic bacteria, can not only attach but also internalize to epithelial cells in a manner different from the control *S. sanguis*.

### Induction of Omp29 surface expression in OmpA-deficient *E. coli*

**Figure 2:**
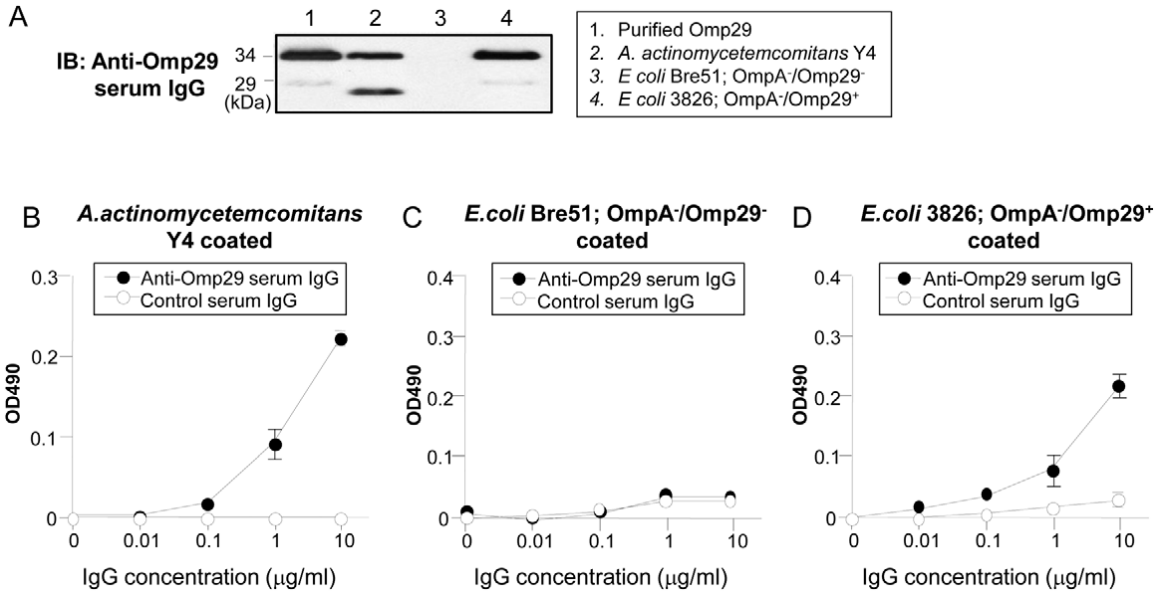
Induction of Omp29 surface expression in OmpA-deficient *E. coli*. (A) Using Western blot analysis, anti-Omp29 serum IgG was reacted to the purified Omp29, whole *Aa* Y4, *E. coli* Bre51 and *omp34* gene-transfected *E. coli* Bre51. The heat-modifiable property of Omp29 resulted in two distinct migration patterns at 29 kD and 34 kD. (B–D) The formalin-fixed whole *Aa* Y4 (B), formalin-fixed whole *E. coli* Bre51 (C) or *E. coli* 3826 (D) was reacted with anti-Omp29-specific IgG and control serum IgG at various concentrations as indicated on the x-axis. The antibody binding to each fixed bacterial antigen was detected with goat anti-mouse IgG-biotin (×10,000), followed by HRP-conjugated avidin (×40,000). Colorimetric reactions were developed with o-Phenylenediamine in appropriate buffer. The optical density (OD) of each well of ELISA plate was measured at 490 nm. The results are expressed as mean OD ± SD of triplicate wells.

Based on the Western blot analysis, the polyclonal anti-Omp29 IgG demonstrated two bands of purified Omp29, *Aa* Y4 and *E. coli* 3826 (Fig. 2A, lane 2 and 4), but not *E. coli* Bre51 (Fig. 2A, lane 3) which lacks both OmpA and Omp29. The heat-modifiable property of Omp29 resulted in two distinct migration patterns (29 and 34 kDa) in an SDS-PAGE carried out in a reductive condition [11], [15]. Binding activity of anti-Omp29 IgG to fixed whole bacteria was also examined by ELISA (Fig. 2B–2D). Anti-Omp29 IgG reacted to fixed *Aa* Y4 and *E. coli* 3826 in a dose-dependent manner as compared to control IgG that showed no distinctive binding activity to the same battery of antigens (Fig. 2B and 2D). Neither anti-Omp29 serum IgG nor control serum IgG reacted to the OmpA deletion mutant *E. coli* Bre51 (Fig. 2C). These results indicated that *E. coli* 3826, but not *E. coli* Bre51, expresses Omp29 on its surface.

### Inhibition of *Aa* Y4 entry into OBA9 by anti-Omp29 IgG antibody

**Figure 3:**
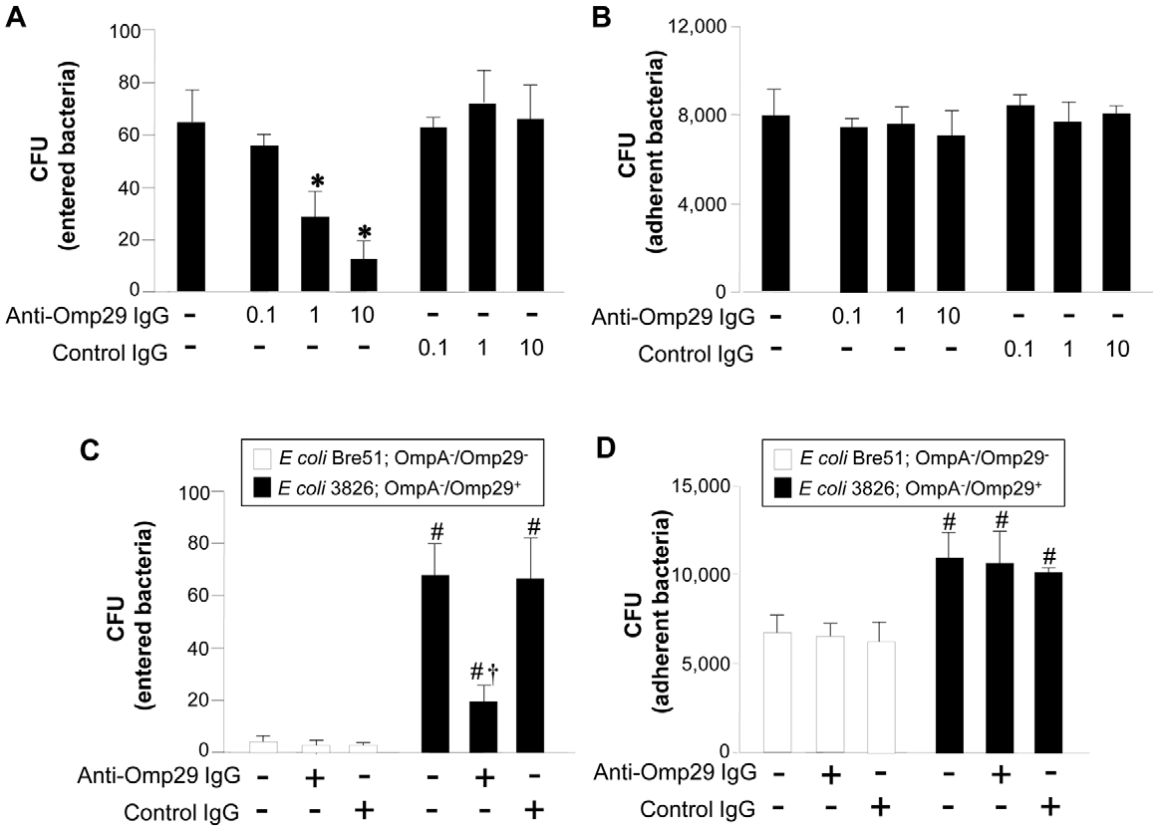
Anti-Omp29 IgG antibody inhibits bacterial entry, but not adhesion, to OBA9. Live *Aa* Y4, Omp29^−^/OmpA^−^ *E. coli* strain Bre51 or Omp29^+^/OmpA^−^ *E. coli* strain 3826 harvested at the mid-log phase was incubated with confluent OBA9 in a 96-well plate (5.5×10^5^ bacteria/100 µl/well) in the presence of anti-Omp29 IgG antibody or control serum IgG. For *E. coli* strains, a dose of anti-Omp29 serum IgG (10 µg/ml) or control serum IgG (10 µg/ml) was applied to each well. After 4 hours of incubation, entry (A) or adhesion (B) of *Aa* Y4, or entry (C) or adhesion (D) of Omp29^−^/OmpA^−^ *E. coli* strain Bre51 or Omp29^+^/OmpA^−^ *E. coli* strain 3826 to OBA9 was measured. Each column represents mean CFU ± SD of three wells. One representative result from at least 3 different experiments is shown. *, Significantly lower than *Aa* Y4 incubated in medium alone (without antibody) or control IgG by Student's *t* test (*P*<0.05). #, Significantly higher than *E. coli* Bre51 entry or adhesion to OBA9 in the absence of antibody by Student *t* test (*P*<0.05). †, Significantly lower than the entry of *E. coli* 3826 incubated in medium alone (without antibody) or control IgG by Student *t* test (*P*<0.05).

The entry or attachment of *Aa* Y4 to OBA9 was examined in the presence or absence of anti-Omp29 IgG or control IgG (Fig. 3). Anti-Omp29 IgG inhibited bacterial entry (Fig. 3A), but not attachment (Fig. 3B), in a dose-dependent manner. Control IgG did not affect the entry (Fig. 3A) or adhesion (Fig. 3B) of *Aa* Y4. Polyclonal mouse IgG antibody to whole *Aa* inhibited both entry and adhesion of the bacteria to OBA9 (data not shown). These results suggest that Omp29 is associated with *Aa* internalization, but not adhesion, to gingival epithelial cells.

### Omp29 increases the entry of OmpA^−^ *E. coli* into OBA9

The role of Omp29 in bacterial entry into OBA9 was further examined using *E. coli* 3826 (Omp29^+^/OmpA^−^) and *E. coli* Bre51 (Omp29^−^/OmpA^−^). *E. coli* 3826 entered into OBA9 22-fold more than *E. coli* Bre51 (Fig. 3C). The entry of *E. coli* 3826 into OBA9 was significantly inhibited by anti-Omp29 IgG, but not by control IgG. Although the ability of *E. coli* 3826 to attach to OBA9 was slightly higher (1.4-fold) than *E. coli* Bre51, anti-Omp29 IgG had no effect on the adhesion of either *E. coli* 3826 or *E. coli* Bre51 to OBA9 (Fig. 3D).

### Omp29 up-regulates F-actin formation in OBA9, which results in induction of *Aa* Y4 entry into OBA9

**Figure 4:**
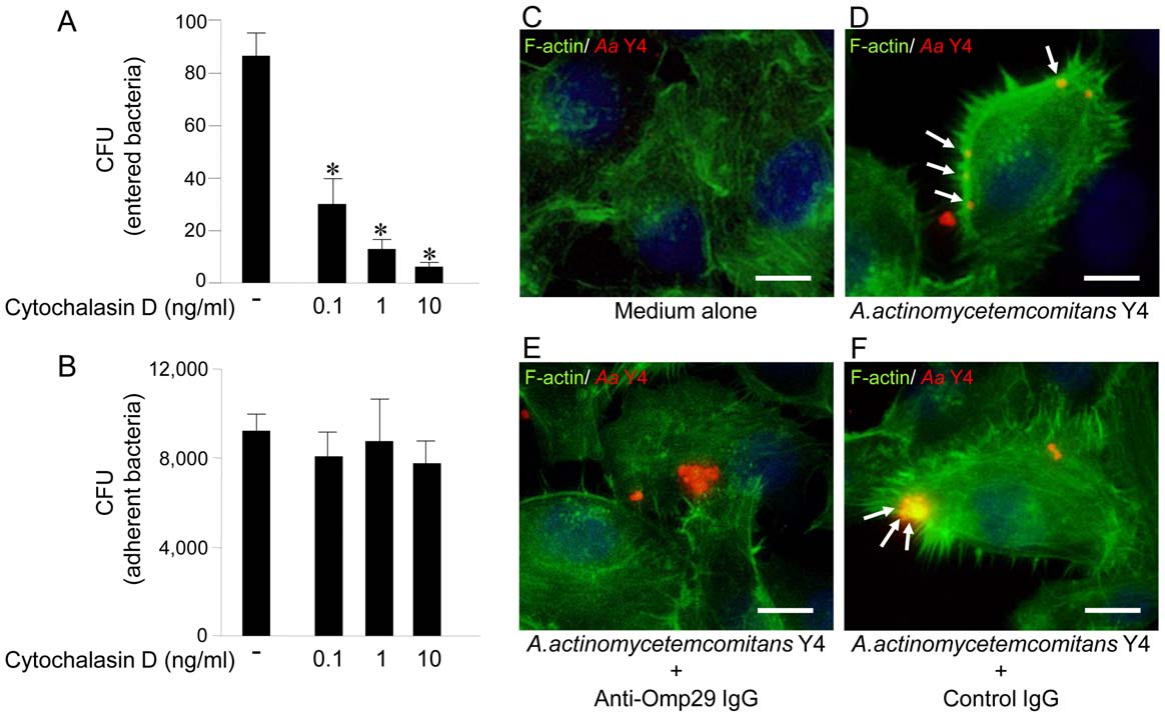
Bacterial Omp29 up-regulates F-actin rearrangement in OBA9, which results in induction of *Aa* Y4 entry into OBA9. (A and B) *Aa* Y4 harvested at mid-log growth was incubated with confluent OBA9 cells (5×10^5^ bacteria/100 µl/well) in the presence or absence of cytochalasin D (F-actin rearrangement inhibitor) by the indicated concentration in the figures. The number of bacteria entered or adherent to OBA9 cells was shown (A, bacterial entry; B; bacterial attachment). *, Significantly lower than control medium alone by Student's *t* test (*P*<0.05). (C–F) After pretreatment of sub confluent OBA9 cells with or without anti-Omp29 IgG (10 µg/ml) or control non-immunized IgG (10 µg/ml) for 15 min, OBA9 cells were co-cultured with *Aa* Y4 (1×10^7^ bacteria/2 ml/dish) in the antibiotics-free K-SFM for 1 hr. F-actin (green) and *Aa* Y4 (red) were observed using immunofluorescence microscopy in OBA9 cells. White arrow indicates co-localization (yellow) of F-actin and *Aa* Y4. (C) medium alone, (D) *Aa* Y4, (E) anti-Omp29 IgG and *Aa* Y4, and (F) control IgG and *Aa* Y4. Scale bar: 10 µm.

**Figure 5:**
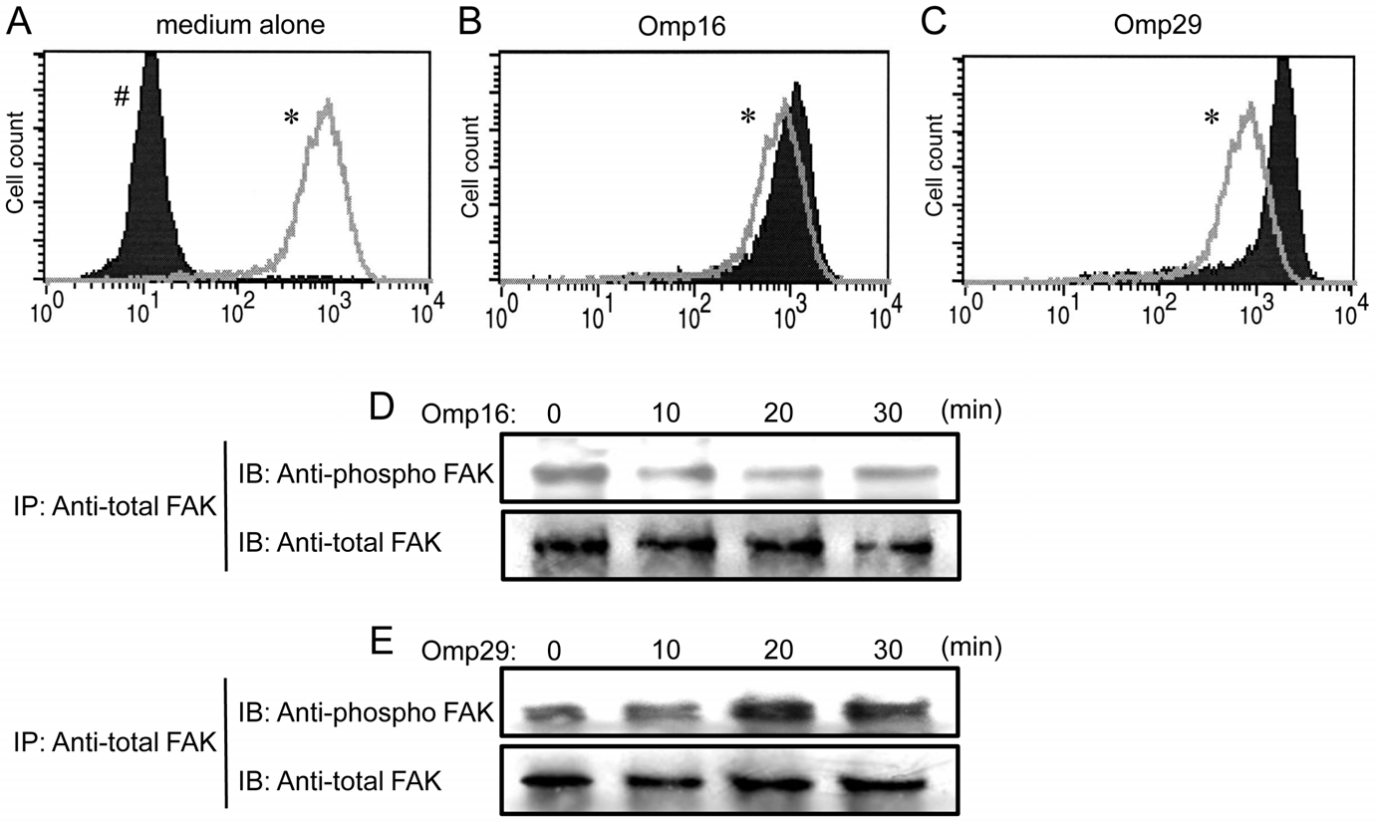
Omp29 induces actin rearrangement and FAK phosphorylation in OBA9. (A–C) The confluent OBA9 cells in the tissue culture flask were incubated for 1 hour in medium alone (A) or in the presence of Omp16 (10 µg/ml) (B) or Omp29 (10 µg/ml) (C). In order to stain F-actin, OBA9 cells removed from culture plate by trypsin-EDTA were permeabilized and stained with FITC-Phalloidin. (A) The solid histogram # shows the non-stained OBA9 and the open histogram in gray line * indicates the FITC-Phalloidin staining of OBA9. (B and C) The open histogram * indicates the FITC-Phalloidin stained OBA9 incubated with medium alone and the solid histograms display the staining of OBA9 stimulated with Omp16 (B) or Omp29 (C), respectively. (D and E) After stimulation of OBA9 cells with Omp16 (10 µg/ml) (D) or Omp29 (10 µg/ml) (E) for the time periods shown in the figure, whole cellular proteins dissolved in lysis buffer were immuno-precipitated with anti-total FAK antibody bound to GammaBind Plus Sepharose beads. The proteins pulled down with the beads were blotted onto NC membrane and reacted with anti-total FAK antibody or anti-phospho FAK antibody using Western-blot method.

A previous study reported that *Aa* strains utilize either actin-dependent or actin-independent mechanisms to penetrate KB cells [21]. Since we wanted to determine if *Aa* Y4 could enter OBA9 by actin rearrangement, the entry and attachment of *Aa* Y4 to OBA9 were examined in the presence or absence of cytochalasin D, an F-actin rearrangement inhibitor (Fig. 4A and 4B). Cytochalasin D inhibited the entry of live *Aa* Y4 into OBA9 in a dose-dependent manner (Fig. 4A), but it did not affect the attachment of live *Aa* Y4 to OBA9 cells (Fig. 4B). In order to determine if bacterial Omp29 regulates F-actin formation and bacterial invasion, OBA9 cells were co-cultured with *Aa* in the presence or absence of anti-Omp29 IgG or control IgG for 1 hr, and the F-actin and *Aa* were visualized by immunofluorescenses (Fig. 4C–4F). Compared to the OBA9 cells treated with medium alone that showed modest level of diffuse F–actin expression (green fluorescent; Fig. 4C), *Aa* Y4 (red fluorescent) applied to OBA9 cells was found co-localized with F–actin which showed remarkably increased level of polymerization (Fig. 4D), indicating F-actin rearrangement was induced in conjunction with bacterial entry. Importantly, anti-Omp29 IgG treatment abrogated such co-localization of *Aa* Y4 and F-actin as well as the increased level of F-actin polymerization (Fig. 4E), whereas increased F-actin polymerization and co-localization with *Aa* were not affected by the presence of control IgG (Fig. 4F). Next, to investigate whether purified Omp29 induce F-actin formation, confluent OBA9 cells were incubated in the presence or absence of 10 µg/ml of purified Omp16 or Omp29 (10 µg/ml, respectively) for 1 hr, and the cytoplasmic content of F-actin was determined by flow cytometry (Fig. 5A–5C). OBA9 incubated with Omp29 showed notable up-regulation of F-actin content (Fig. 5C) compared to OBA9 incubated in medium alone (Fig. 5A) or OBA9 reacted with Omp16 (Fig. 5B), respectively. These results suggested that *Aa* Y4 induces actin rearrangement via Omp29 which, in turn, leads the entry of *Aa* Y4 into OBA9 cells.

### Omp29 induces FAK phosphorylation

It is well described that focal adhesion kinase (FAK) is an important signaling switchboard in the course of actin rearrangement triggered by the stimulation of cell surface adhesion molecules [22], [23]. To examine the effect of Omp29, or control Omp16, on the FAK phosphorylation in gingival epithelial cells, OBA9 cells were reacted with Omp16 or Omp29 for various periods. Subsequently, whole FAK-protein captured by antibody-mediated immuno-precipitation was blotted onto nitrocellulose membrane and reacted with anti-phosphorylated FAK antibody (Fig. 5D and 5E). The Omp29 stimulation clearly up-regulated the phosphorylation of FAK in OBA9 cells in a time-dependent manner (Fig. 5E), while Omp 16 did not induce phosphorylation of FAK (Fig. 5D).

### Omp29, but not Omp16, evokes internalization in OBA9

**Figure 6:**
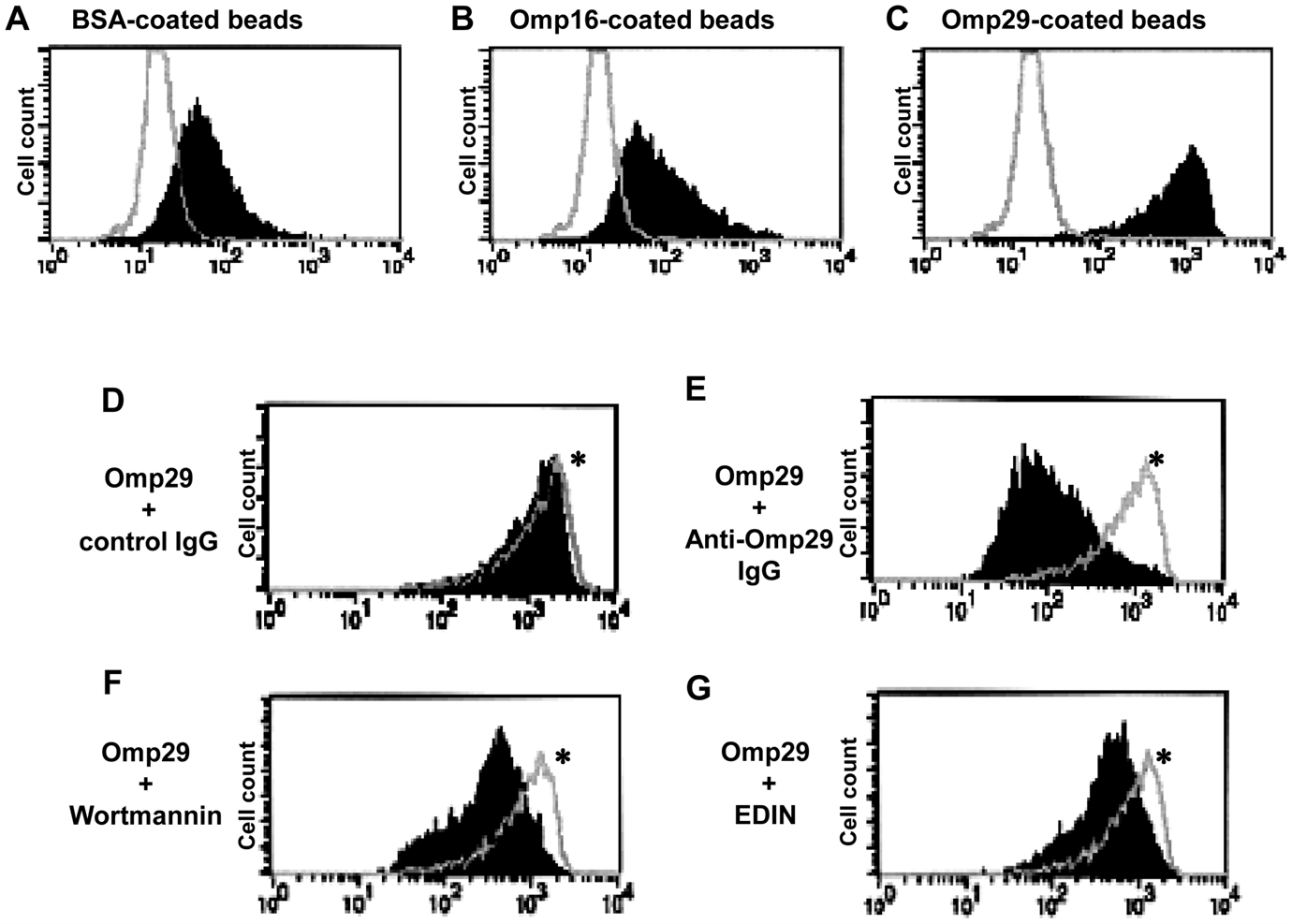
Influence of Rho GTPase inhibitor and PI-3-kinase inhibitor on internalization of Omp29-coated beads into OBA9. (A–C) Confluent OBA9 cells cultured in tissue culture plate were incubated with or without BSA-coated fluorescent Latex beads (FLB) (A), Omp16-coated FLB (B), or Omp29-coated FLB (C) for 4 hours. The open histogram indicates non-treated OBA9 and the solid histogram display the internalization of indicated FLB, respectively. (D–G) Omp29-coated FLB were incubated with OBA9 cells for 4 hours in the presence or absence of control serum IgG 10 µg/ml (D), anti-Omp29 serum IgG 10 µg/ml (E), Wortmannin 10 nM (F), or EDIN 100 ng/ml (G). Open histogram * indicates the internalization of Omp29-coated FLB in OBA9 incubated with medium alone. The solid histogram display the incorporation of Omp29-coated FLB treated with IgG or inhibitory agents, respectively. Anti-Omp29 serum IgG, Wortmannin and EDIN inhibited 97%, 73% and 66% of Omp29-coated FLB internalization into OBA9 cells, as determined by mean fluorescent intensity.

To confirm whether Omp29 triggers internalization in OBA9, fluorescent Latex beads coated with purified Omp29, Omp16 or BSA were incubated with OBA9, and the beads internalized into OBA9 were analyzed by flow cytometry (Fig. 6). Omp29-coated beads were incorporated into OBA9 at a greater magnitude than BSA-coated beads or Omp16-coated beads (Fig. 6A, B and C). BSA-coated beads also entered OBA9 (Fig. 6A), but only slightly. To explain this, it is reported that rat gingival junctional epithelium can uptake topically applied horseradish peroxidase or colloidal gold-labeled concanavalin A [24], [25]. Thus, the uptake of latex beads by BSA appears to have resulted from the intrinsic characteristics of epithelial cells. Taken together, these findings indicated that Omp29 triggers internalization in OBA9.

### FAK downstream signaling, PI3-kinase and Rho GTPase are involved in the internalization of Omp29-coated beads and *Aa*

**Figure 7:**
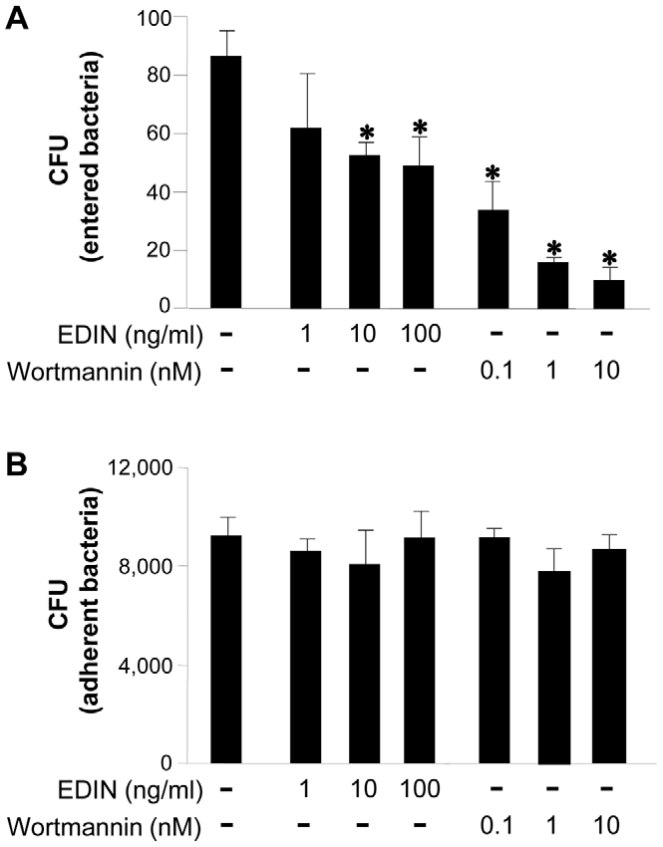
Influence of Rho GTPases inhibitor and PI-3-kinase inhibitor on bacterial entry and attachment to OBA9. *Aa* harvested at mid-log growth phase was incubated with confluent OBA9 cells (5×10^5^ bacteria/100 µl/well) in the presence or absence of Wortmannin or EDIN by the indicated concentration in the figures. The number of bacteria that entered or adherent to OBA9 cells is shown (A, bacterial entry; B, bacterial attachment). *, Significantly lower than control medium alone by Student's *t* test (*P*<0.05).

It is well recognized that PI3-kinase and Rho GTPase are downstream targets of FAK [26], and we found in this study that Omp29 induced the phosphorylation of FAK (Fig. 5). Therefore, to examine whether Omp29-mediated internalization of *Aa* Y4 in gingival epithelial cells involves these signal transducers, inhibitory assays were performed using flow cytometry. Omp29-coated fluorescent Latex beads were incorporated into OBA9 at a greater magnitude than BSA-coated beads or Omp16-coated beads (Fig. 6A, B and C). BSA-coated beads also entered OBA9 (Fig. 6A), but only slightly. The internalization of Omp29-coated beads was inhibited by anti-Omp29 serum IgG (Fig. 6E), but not by control serum IgG (Fig. 6D). Wortmannin (PI3-kinase inhibitor; 6F) and EDIN (signal transduction inhibitor Rho GTPases; 6G) also inhibited internalization of Omp29-coated beads into OBA9. Both inhibitors, Wortmannin and EDIN also interrupted the entry of live *Aa* Y4 into OBA9 in a dose-dependent manner (Fig. 7A), whereas these inhibitors did not affect the attachment of live *Aa* Y4 to OBA9 cells (Fig. 7B). These findings suggested that Omp29-induced bacterial internalization is involved in PI3-kinase and Rho GTPase signaling in OBA9 cells.

## Discussion

It is proposed that at least four different steps are required for *E. coli* K1 invasion into brain endothelial cells: 1) initial binding, 2) tight binding/invasion, 3) host cell cytoskeletal rearrangement, and 4) intercellular trafficking [27]. Each step of the bacterial invasion event is engaged by distinct bacterial components [27]. We found that *Aa* Omp29 is associated with bacterial entry into gingival epithelial cells by up-regulation of F-actin rearrangement. Therefore, Omp29 appears to operate in accordance with step 3 of this multiple-step paradigm, only substituting *Aa* for *E. coli* invasion.

*E. coli* OmpA, a homologue of Omp29, binds to gp96 expressed on endothelial cells and induces actin cytoskeletal rearrangements [28]. In our study, Omp29 induced F-actin rearrangement and the resulting bacterial internalization in OBA9 cells (Fig. 4). On the other hand, we also previously reported that *Aa* Omp100 is associated with adhesion of *Aa* to gingival keratinocytes [10]. According to our homology search, Omp100 showed high similarity to *Yersinia enterocolitica* YadA [11] that is associated with Yersinia adhesion to extracellular matrix proteins [29] and fibroblasts [30]. Coincidently, Li et al. also reported that a gene locus of *Aa* encoding putative invasion-related protein is homologous to *Yersinia enterocolitica* YadA [31]. However, in the latter study, the gene encoding the OmpA/Omp29 family of molecules was not detected in the invasion-related gene locus of *Aa*. The different roles played by Omp100 and Omp29 in bacterial entry were distinguished by bacterial entry inhibition assays using specific antibody to each molecule, i.e., anti-Omp29 antibody inhibits the entry, but not the adhesion, of *Aa* (Fig. 3), whereas anti-Omp100 antibody inhibits both adhesion and entry of *Aa* to gingival epithelial cells [10]. These findings led to the new hypothesis that initial bacterial adhesion mediated by Omp100 allows Omp29 binding to its putative ligand expressed on gingival epithelial cells. Additional study will be required to clarify how Omp100 and Omp29 are engaged in the distinct steps of bacterial internalization, i.e., adhesion and entry, either independently or together. For example, the development of *E. coli* Bre 51 mutant strain that expresses both Omp100 and Omp29, or Omp100 alone, would provide the clue to evaluate this new hypothesis.

It is well recognized that FAK plays an important role in the course of actin rearrangement triggered by cell surface adhesion molecules [22]. Furthermore, the signaling cascade initiated from FAK activation is documented in the mechanism underlying cytoskeletal fiber rearrangement that, in general, involves PI3-kinase and Rho GTPase [23]. In the present study, Omp29 induced F-actin rearrangement which resulted in the bacterial internalization in OBA9 (Fig. 4) and the up-regulation of FAK phosphorylation within 30 min (Fig. 5). In addition, the bacterial internalization into OBA9 was remarkably abrogated by PI3-kinase or Rho GTPase inhibitor (Fig. 7). These findings indicated that *Aa* Omp29 may induce F-actin rearrangement by activation of FAK-PI3kinase or -Rho-GTPase signaling cascade, which, in turn, would result in OBA9 bacterial internalization. It is possible that such an early cellular response occurs only if it is independent from gene transcription. When we used the RNA synthesis inhibitor actinomycin D to test this possibility, it neither altered the entry of *Aa* nor the internalization of Omp29-coated beads into OBA9 (data not shown), further supporting the argument that Omp29-mediated actin rearrangement does not require gene transcription.

Penetration of bacterial protein into host tissue is a necessary step toward inducing adaptive immune response, such as production of antigen-specific IgG. Remarkably elevated Omp29-specific IgG in the serum of patients with localized aggressive (juvenile) periodontal disease [32] suggests that Omp29 is one of the major antigens penetrating host tissue, and, as such, it is recognized by lymphocytes. Using a rat periodontal disease model, we demonstrated that systemic adoptive transfer of Omp29-specific Th1 cells, along with the local gingival injection of Omp29, can result in RANKL-dependent periodontal bone loss, whereas adoptive transfer of the Th1 cells alone without local antigen injection did not induce bone resorption [33], [34]. Furthermore, cultured rat gingival epithelial cells could process the internalized formalin-killed *Aa* and present Omp29 antigen to Th1 cells, which resulted in their activation and proliferation [35]. These studies support that bacterial entry into the host tissue or cell is requisite to elicit the host destructive Th1 immune responses, because, without internalization of bacterial antigen in the host, immune response can not be induced. The results from the present study, together with our previous demonstrations of Omp29-mediated immune intervention in rat periodontal destruction [33], [34], [36], strongly suggest that penetration of Omp29 into gingival epithelial cells plays a pathogenic role in the course of periodontal disease caused by *A. actinomycetemcomitans*.

Taken together, the results obtained in this study elucidated that *Aa* Omp29 acts on gingival epithelial cells to cause F-actin rearrangement via FAK signaling cascade, which results in the uptake of *Aa* into human gingival epithelial cells. Future study will be required to identify the receptor for Omp29 expressed on gingival epithelial cells. Such studies will lead to a better understanding of the molecular mechanism that up-regulates bacterial uptake by gingival epithelial cells and thereby provide for the development of novel preventive or therapeutic regimens in the treatment of periodontal disease.
